# Introduction for the Special Issue: Statistical Fluid Dynamics

**DOI:** 10.3390/e24060782

**Published:** 2022-06-01

**Authors:** Amine Ammar, Francisco Chinesta, Rudy Valette

**Affiliations:** 1 ESI Chair @ LAMPA, LAMPA, Arts et Metiers Institute of Technology, HESAM Université, CEDEX 01, F-49035 Angers, France; 2 ESI Chair @ PIMM, PIMM, CNRS, CNAM, Arts et Metiers Institute of Technology, HESAM Université, F-75013 Paris, France; francisco.chinesta@ensam.eu; 3 MINES-ParisTech, CEMEF, PSL Research University, F-06904 Sophia Antipolis, France; rudy.valette@mines-paristech.fr

Characterizing complex material consists in establishing the relationship between flow rheology during forming processes and the induced micro-structural state that affects directly the final mechanical properties of the formed parts. It is necessary that research activities reach to address the coupling between forming process and mechanical performances (e.g., fatigue or reliability). Even if in this issue we put the main attention on the fluid dynamic part, the research activity today must cover the life cycle from individual kinematics of particles to the mechanical properties of formed parts. Some points of illustration are quoted here on a non-exhaustive basis:

- Individual kinematics of one fiber or particle in non-Newtonian flow and consideration of hydrodynamic interactions remains today an open subject experimentally as well as numerically (using direct approaches). No accurate analytical characterizations have been done in non-Newtonian fluids in infinite domains. A specific attention must be considered for viscoelastic materials. Such a material is usually encountered during forming processes of injection molding or thermoplastics extrusion.
- After this first step, one must be able to make statistical transitions in order to predict the orientation distribution of a suspension of particles. This step suppose that the individual kinematics has been well established and must take into account particles interactions in order to predict different phenomena such as aggregation.
- Composites involve in their liquid or solid state a significant fluctuation of physical properties due to their heterogeneity. In order to get a fine description, numerical models use generally an excessive computing time and requires a high capacity of storage. This is a consequence of the high number of degrees of freedom requested to correctly describe the physical properties. It will then be necessary to use some approaches that reduce the numerical cost, such as the POD (Proper Orthogonal Decomposition) or the PGD (Proper Generalized Decomposition). These methods will make simulations of complex flows easier. The main difficulty consists in predicting correctly the microscopic state that affects directly the final properties. Two modeling scale are to take into account: the first one is related to the global dimension of the flow providing velocity field, thermal distribution, pressure and other macroscopic fields. The second one is related to the orientation of fibers, the conformation of molecules, etc.
- Establishing distribution models is an essential information for reliability models. Reliability of composites have to be studied through the part life cycle. This requires reproducing (numerically and experimentally) the succession of loadings inducing material damage. Modeling aspects have to be oriented to establish the tools allowing to predict failure probability. These tools could be built by using the statistical information previously calculated.
- Next, fatigue and damage models using internal variables have to be related to the induced microstructure. Indeed, modeling of fatigue is constraint by the CPU time and requires to simulate a very high number of cycles. Two strategies are possible in order to circumvent the difficulty related to the computational cost of the temporal scale: (i) the first one consists in making a decomposition of the time dimension into two dimensions, where the first one is related to a finite small number of cycles and the other one is related to a global evolution of the internal variables. (ii) And the second strategy consists to come back to POD-like techniques which are suitable for extracting modes in cyclic behavior.
- These approaches must be associated to homogenization procedures for complex materials. A specific knowledge of space scales transitions and the relationship with the Representative Elementary Volume (REV) during the forming process or the mechanical loadings, is an essential information for using composites material during their life cycle. Fatigue models in direct simulations could be compared to fatigue model with homogenized variables.
- Machinability of complex materials is also an interesting subject. Modeling the cutting process and the confrontation with experimental measures could give an idea to bring a multi-physic comprehension of chip formation and the tool/workpiece interaction by adopting finite element approaches and methodologies. Microscopic state is determinant in these conditions.

In all this sequence just described the challenge in the flow phase consists to establish the relationship between the flow rheology during the flowing process and the state of the induced micro-structure that directly affects the quality of the obtained mechanical parts. This characterization should take into account the multi-scale description of the continuum matter. Many developments are required. One of them consists in identifying experimentally, numerically, and analytically the laws that govern each scale rigorously. Another challenge consists in developing numerical techniques that allow addressing a detailed description of the physical laws involving a large number of degrees of freedom. Development and control of advanced numerical techniques and experimental observation is essential to predict accurately and with a lowest cost the state of matter and the resulting properties.

Achieving the goal of modeling micrometric and nanometric suspensions remains a major issue. This help to master in a controlled way the mechanical, thermal, and electrical properties, among others, of the suspensions and then of the resulting product when considered in material forming, flow of heat transfer fluids or other applications. In some cases, they can contribute to improve the performance of energy transport. An optimal use of these products is based on an accurate prediction of the flow-induced properties of the suspensions and consequently of the resulting products and parts.

The scientific issues to solve are mainly related to the prediction of the behavior evolution. Particles suspended in a viscous medium tend to modify the behavior. The final properties of the resulting microstructured fluid or solid become radically different from the simple mixing rule. There are numerous works addressing homogenization strategies for systems consisting of perfectly of dispersed particles in a matrix. However, in most cases, particles aggregate or sediment, or exhibit strong induced anisotropy. The microscopic description, despite being the finest one, is too heavy from both computational and experimental points of view. For this reason, coarser descriptions are sometimes preferred. Even if they are less accurate, they lead to faster simulations.

Considering the general behavior of suspensions, viscoelastic fluids or complex flows, two levels of description are relevant: a level related to the overall kinematics and a level associated with the material point in the microscopic scale [1].

Taking into account the state of microscopic structure can be done at different scales. For some behaviors such as fibers, differential approximation requires a closure relationship [2]. Unfortunately, in most cases (except for some special ones), there is no equivalence between the constitutive laws and the microscopic definition of the structure. A microscopic simulation at the scale of the kinetic theory is then required.

The most common technique for kinetic theory problems is the stochastic approach. A lot of work has been done on different models of kinetic theory (dumbbell models, fibers, polymer melts…) see, for example [3,4].

For higher dimensions (much higher than three) stochastic methods become limited. In the few studies that we find in the literature on the simulation of this type of problem, the authors use a discrete approach (Brownian or with Monte Carlo) that involves the use of a large number of particles. In very special cases, the probability distribution evolution can be expressed as N evolution problems of N different functions with a vectorial change of variable.

The framework for these problems requires the development of specific numerical techniques applicable for problems with large numbers of degrees of freedom.

The difficulty of multidimensional models resolution is related to the proposal of new appropriate strategies able to circumvent the curse of dimensionality. One possibility lies in the use of sparse grids [5]. However, as argued in ref. [6], the use of sparse grid is restricted to models with moderate multidimensionality (up to 20). Another technique able to circumvent, or at least alleviate, the curse of dimensionality consists in using a separated representation of unknown fields (see ref. [7]. for some numerical elements on this topic).

The question of multidimensionality has also been subject of works related to the space-time separated representation. In fact, such decompositions were proposed many years ago by Pierre Ladeveze as an ingredient of the powerful non-linear-non-incremental LATIN solver that he proposed in the 80s. During the last twenty years many works were successfully accomplished by the Ladeveze group. The interested reader can refer to [8] and the references therein related to the radial approximation, denotation given to the space-time decomposition in the LATIN framework.

The resolution of problems with analytical solution is rarely possible. Analytical solution is provided only for specific simplified equations. Otherwise, solution is searched for as a discrete form over a given set of points. Once the discrete solution is obtained in these points the continuum solution can be built on using an appropriated interpolation. When model is defined in dimension *D*, and with *N* degrees of freedom in each direction the resolution requests $N^{D}$ discrete points. The difficulty related to the information processing and storage becomes exponentially dependent on the dimension *D*. Beyond the value of *D* equals to 3, standard discretization techniques (such as finite elements, finite differences, or finite volumes methods) suffer from the limitations related to the high number of degrees of freedom.

On this numerical point of view, some contributions have been focused on the development of a new strategy different from the classical based-mesh techniques (FEM, FDM, FVM). The developed method called the PGD (Proper Generalized Decomposition) allows circumventing the curse of dimensionality and allows particularly to solve space-time problem avoiding the use of standard incremental time scheme. It allows more generally to solve problems defined in multidimensional space. The main idea consists to build up the multidimensional solution as a tensor product of functions expressed in lower dimensions.

This strategy was useful for transient problem, but also has proved its robustness for solving micro-macro problems by including configuration and physical spaces in the same discretization. In addition, this strategy has been successfully applied for solving kinetic theory problem when configuration space dimension exceeds the value three. This situation in encountered in the kinetic theory of melt polymer or for bead-spring-chain model for polymer suspension.

The obtained results as well as the potential application are encouraging to carry on the development of this technique and to improve its performance in terms of convergence speed and optimality and to enlarge its application fields.

In addition, considering in general the behavior of statistical fluids, fiber suspensions, polymers suspensions, viscoelastic fluids, we see that there are two levels of flow description:

(i) The first one is a level related to the global flow kinematics variables (such as velocity) requiring a variational formulation of the problem overall the physical domain.
(ii) The second one is a level related to the elementary representative volume which characterizes a state of matter: an orientation or conformation induced by the flow. This condition defines the effect of the microscopic structure of the flow.

Taking into account the state of microscopic structure can be done at different scales. One can use in a first approach a constitutive law (differential or integral) to describe the evolution of the stress tensor characterizing the structure. For some behaviors such as fibers, the constitutive law is written in terms of an approximation introducing a closure relation. Unfortunately, in most cases (excluding some constitutive equation written rigorously), there is no equivalence between the definition of the microscopic structure evolution and the constitutive law. In fact, the approximation introduces some errors. This is particularly the case for fiber suspension where there is a high incidence of the error induced by a closure relation on the orientation tensor when the diffusion parameter is small.

If we wish to describe directly the evolution of the structure, a fine modeling at the microscopic scale reveals itself indispensable.

The way in which we describe a microscopic behavior is based on (i) the kinematics of each particle and (ii) the evolution of a probability distribution on the configuration space of all the particles, also called the probability space. From a probability distribution one can go back to the macroscopic state through a calculation of the stress tensor giving the microscopic contribution. We consider that the kinematics of each particle is given by a hydrodynamic contribution and interactions efforts contribution. Terms arising from Brownian effects are obviously taken into account in the diffusive contribution of the convection-diffusion equation characterizing the evolution of the probability distribution. This equation is the so-called Fokker-Planck equation.

For example, in the context of multi-dumbbells models some contributions have allowed to find a solution of the Fokker-Planck equation as a sum of functions products in the context of the PGD [9]. This also has been done in the case of polymer melts [10].

Such approaches of numerical modeling at small scales also have many advantages in the bio-medical field. Macromolecules such as DNA chains can be modeled with high-dimensional configuration spaces. The difficulty arises in situations in which one wants to lead a macromolecule (pharmaceutical drug for example) in into a pipe of very small size (e.g., a vein) without the use of tools. We should then be able to predict the properties of the velocity field so that the macromolecule gets the desired state.

In the same framework, the kinetic description of the rheology of carbon nanotubes suspensions where the direction and also the aggregation state of the system has been addressed [11].

In carbon nanotubes suspensions one must distinguish the case where the nanotubes are functionalized to prevent their aggregation and the case if they are not. This latter situation is able to lead to their aggregation with significant effects on the rheological properties.

In the case of the functionalized nanotubes, kinetic model has been developed for short suspensions and has been relevant to describe the nonlinear rheology. 

When now we come back to the upper scale of the global flow (in a framework of micro-macro approach), the difficulty lies in predicting the state of micro-structure which affects directly the final properties. We then have a level of modeling on the scale of the geometry of the flow giving kinematics, thermal field, pressure… and a level of modeling of the microscopic characterization, of the state of orientation, of a fiber suspension, or of the conformation of a macromolecule’s population…

To this end, developments are necessary:

- To be able to consider the relevant microscopic information in order to integrate more physical responses such those finely described with molecular dynamics.
- To integrate the micro-macro coupling—for which we must create the required techniques in adequacy with a rapid integration of microscopic behavior (finely described at the representative elementary volume of flow with the probability distribution) in a simulation code.

The scope of application of this work is the engineering of complex fluids. Although several studies have been done by substituting the microscopic description by using approximations based on constitutive differential or integral equations, it turns out that the kinematics of the flow is highly affected by the topology of the microstructure; consequently, we have to treat more carefully the microscopic information. The objective is to make the interaction between the kinematics of the flow behavior and the molecular information at the lowest numerical cost. This then requires an appropriate use of specific techniques of model reduction to adequately describe the probability distribution on a hyperspace resulting from a combination of physical space, the configuration space and the temporal dimension.
